# Commentary: Principles, Approaches and Challenges of Applying Big Data in Safety Psychology Research

**DOI:** 10.3389/fpsyg.2019.02801

**Published:** 2019-12-10

**Authors:** Davide Giusino, Federico Fraboni, Marco De Angelis, Luca Pietrantoni

**Affiliations:** ^1^Department of Psychology, University of Bologna, Bologna, Italy; ^2^Interdepartmental Center for Industrial Research in Advanced Mechanical Engineering Applications and Materials Technology, University of Bologna, Bologna, Italy

**Keywords:** big data, multidisciplinary psychology, big data of whatever psychology, big data of safety psychology (BDSP), psychology applied to big data

## Introduction

This commentary builds upon the recent theoretical paper by Kang et al. (2019) to advance the debate currently going on about psychology and big data. The aim is to discuss the feasibility of extending the conceptualization proposed by the authors—i.e., Big Data of Safety Psychology (BDSP)—to other branches of psychology going beyond the only safety domain, ultimately pointing out a *big data of whatever psychology* scenario. This will lead to suggest a perspective enrichment from a solely *big data applied to psychology* paradigm toward a much less advocated *psychology applied to big data*.

## Big Data of Whatever Psychology

Big Data are generally defined as data being high in volume, velocity, and variety. That is, a huge amount of data is produced at an inedited fast pace coming from broadly diverse sources. Big data are characterized by varying quality (*veracity* issues) and are usually unstructured (raw digital information like texts or images) vs. structured (data traditionally representable on statistical software spreadsheets like numbers or Likert-type measurements). Big Data result from a global *datafication* phenomenon that is impacting contemporary human everyday life (Chen and Wojcik, 2016). Indeed, nowadays people are constantly using various types of online and offline digital ICTs—especially social media—which generate great quantities of data.

Although not free from pitfalls, Big Data are offering benefits to many academic and industrial sectors. This is because information can be inferred, and predictions made, based on the inductive observation of real-world behavioral patterns displayed by unprecedently large datasets. In the history of psychology, the official turning point in big data uptake can be traced to the 2016 *Psychological Methods* special issue entirely devoted to such topic (Harlow and Oswald, 2016). From there on, big data approaches to psychology have become increasingly popular (Jones, 2017), and several scholars have initiated both theoretical and empirical investigation entailing the use of big data within different sub-disciplines of psychology.

Among these, Kang et al. (2019) introduced the BDSP concept to hold how big data adoption and utilization may positively affect workplace health and safety. They define BDSP as “structured, semi-structured, and unstructured datasets formed by psychological index parameters and behavior, which provide potential and valuable psychological knowledge and rules to solve the psychological issues related to safety with the help of big data technology” (p. 3). Of course, safety is an extremely interesting and practically relevant field for big data implementation as it is aimed at managing risks and preventing accidents. Nevertheless, the paper provides input to wonder why we should limit our scope to the safety domain and not including other ones into the *big data of psychology* discourse. Examples of big data applications are identifiable in numerous areas of psychology, such as organizational (Guzzo et al., 2015; Tonidandel et al., 2016), educational (aka learning analytics; Watson and Christensen, 2017; Maldonado-Mahauad et al., 2018; Viberg et al., 2018; Elia et al., 2019; Shorfuzzaman et al., 2019), marketing (Hopp and Vargo, 2017; Matz and Netzer, 2017; Erceg et al., 2018; Ibrahim and Wang, 2019), personality (Bleidorn et al., 2017; Boyd and Pennebaker, 2017; Gerlach et al., 2018; Hinds and Joinson, 2019), emotion (aka affective computing; D'Mello et al., 2018; Chatterjee et al., 2019; Gruda and Hasan, 2019), psycholinguistics (Ridgeway et al., 2017; Johns, 2019; Luo et al., 2019), clinical (Anestis et al., 2016; Russ et al., 2018), cognitive (Medina and Fischer-Baum, 2017; Bhatia and Walasek, 2019), community (O'Brien, 2016), group (Guadagno et al., 2018), music (Greenberg and Rentfrow, 2017), political (Ma-Kellams et al., 2018), and positive psychology (Luhmann, 2017; Yaden et al., 2018). Additionally, the representation of BDSP as being the intersection of safety science, data science, and psychology, seems to equally fit other psychology branches. For instance, we might say that *big data of organizational psychology* constitute the link between organization science, data science, and psychology. As well, one may state that *big data of political psychology* connect political science, data science, and psychology. Basically, it will suffice to replace the first term of the equation. Finally, the seven listed characteristics, along with the 15 types of BDSP, are also easily transferable to different psychological sub-domains. This line of reasoning leads us to conclude that we are actually in front of a *big data of whatever psychology* (BDWP) landscape.

## From “Big Data Applied to Psychology” TOWARD “Psychology Applied to Big Data”

All BDWP literature outputs show one common feature. They all encompass big data as a tool or instrument at the disposal of psychological disciplines. So, no more doubts are around big data usefulness in psychology—and for sure we need further methodological developments and researchers' upskilling to exploit it (Liem et al., 2018; Bleidorn and Hopwood, 2019). Today, the question is no longer whether big data should be used in psychological applications, or what the role of big data in psychology could be.

Therefore, new conceptualization needs regarding the relationship between psychology and big data are stimulated. As a call for discipline self-reflection, we suggest that the question is now becoming what the role of psychology might become in a big data era. This would imply a paradigm shift from *big data applied to psychology* toward *psychology applied to big data*. For instance, it is recognized that big data are associated with individual, cultural, organizational, structural, and technological barriers hindering their acceptance. These entail concerns about privacy and personal information confidentiality (Fast and Jago, 2019), lack of IT infrastructure readiness, and poor analytic skills (Alharthi et al., 2017). These factors can determine resistance to their adoption (Raguseo, 2018), increase people's skepticism toward this technology (Van Rijmenam, 2014), and thus undermine its potential benefits.

How are psychological disciplines going to address similar issues, for example by deploying human-technology interaction frameworks (e.g., Technology Acceptance Model; Davis, 1989) or organizational change management strategies (e.g., Calvard, 2016)? Are psychologists going to only use already available tools or also develop new tailored, targeted ones? Future research can use this kind of questions to start stepping forward within the theoretical, empirical and practical debate about psychology and big data.

## Author Contributions

DG wrote the first draft of the manuscript. FF, MD, and LP contributed to manuscript revision, read, and approved the submitted version.

### Conflict of Interest

The authors declare that the research was conducted in the absence of any commercial or financial relationships that could be construed as a potential conflict of interest.
